# Evaluating the Effectiveness of Insecticides on Spotted Lanternfly *Lycorma delicatula* (Hemiptera: Fulgoridae) in Kiwifruit

**DOI:** 10.3390/insects16090954

**Published:** 2025-09-11

**Authors:** Zi-Jian Song, Yi-Na Bai, Zheng-Yu Luo, Rebecca Burns, Chandan Pal, Feng Zhang, Shu-Sen Shi, Rui Bi, Jin-Ping Zhang

**Affiliations:** 1 Key Laboratory of Soybean Disease and Pest Control, Ministry of Agriculture and Rural Affairs, College of Plant Protection, Jilin Agricultural University, No. 2888 Xincheng Street, Changchun 130118, China; 15523477823@126.com (Z.-J.S.); sss-63@263.net (S.-S.S.); 2 MARA-CABI Joint Laboratory for Bio-Safety, Institute of Plant Protection, Chinese Academy of Agricultural Sciences, No. 2 Yuanmingyuan West Road, Beijing 100193, China; byn2249462@163.com (Y.-N.B.); luozhenggyu@163.com (Z.-Y.L.); f.zhang@cai.org (F.Z.); 3 Zespri International Limited, 400 Maunganui Road, Mount Maunganui 3116, New Zealand; rebecca.burns@zespri.com (R.B.); chandan.pal@zespri.com (C.P.); 4 CAB International, 12 Zhong Guancun South Street, Beijing 100081, China

**Keywords:** spotted lanternfly, insecticide, kiwifruit, pest management

## Abstract

The spotted lanternfly (SLF), *Lycorma delicatula* (Hemiptera: Fulgoridae), has invaded several countries and caused significant losses to agricultural economy, especially in grapes, apples and kiwifruit production. Currently, chemical control is the primary method for managing this pest. In this study, we tested the contact toxicity of bifenthrin and lime sulphur on SLF eggs, as well as the residual toxicity of natural pyrethrins, bifenthrin, and thiacloprid on SLF nymphs. The results showed that bifenthrin, applied at 100 times the label rate, had the highest lethal effect on SLF eggs, with an adjusted mortality rate of 71.8 ± 8.5%. The addition of a penetrant, Engulf, did not further enhance ovicidal efficacy. For nymphs, the residual effect of thiacloprid and bifenthrin at label rates demonstrated 100% mortality within 48 h in laboratory bioassays. Similar efficacies were observed in short-term field experiments. However, the efficacy of bifenthrin declined significantly over time, while thiacloprid provided a long-lasting control, with mortality rates of 72.8 ± 5.9% and 46.7 ± 7.2% at 7 and 14 days, respectively. Although the combination of pyrethrins and mineral oil exhibited limited efficacy, with a 0-day (test immediately after the sprayed branches have dried) mortality rate of 41.5 ± 12.5%, its favourable environmental safety profile makes it a viable option for organic control strategies. In conclusion, bifenthrin at 100 times the label rate was the most effective against SLF eggs, while thiacloprid at label rates provided the longest-lasting control against SLF nymphs.

## Simple Summary

The spotted lanternfly (SLF) is a highly destructive insect that has invaded several countries, causing major economic losses to important crops such as grapes, apples, and especially kiwifruit. As this pest continues to spread globally, countries that are currently free from it, such as New Zealand, need an effective defense plan in case of an invasion. Our study aimed to identify the best tools to fight this pest by testing several chemical controls that are compliant with New Zealand’s safety standards. We tested the efficacy of certain insecticides against SLF eggs by spraying the recommended concentration, as well as 10 times and 100 times the recommended concentration. We also evaluated the short-term and residual efficacy of the recommended concentration on SLF nymphs in both laboratory and field settings. Our results showed that bifenthrin was the most effective at killing the eggs, but only when applied at a very high concentration—100 times the normal rate. For controlling the nymphs, the recommended concentration of thiacloprid proved superior, as it not only showed quick action/effect but also continued to protect the plants for at least two weeks. Our findings provide a potential, ready-to-use strategy against SLF. This knowledge allows for a rapid and effective response to manage a potential spotted lanternfly outbreak, helping to safeguard a vital part of the agricultural economy.

## 1. Introduction

The spotted lanternfly (SLF), *Lycorma delicatula* White (Hemiptera: Fulgoridae), is an invasive insect pest of global significance that can cause significant damage to a wide range of agricultural and horticultural crops [1]. In Japan, reports about SLF can be traced to the 1930s [1]. SLF populations have been documented in China and Korea [1], and it was discovered in Berks County, Pennsylvania in the United States in 2014 [2]. Then successfully established itself in many states of USA, spanning New England, the Mid-Atlantic, the Midwest, and the Pacific areas [3,4]. Currently, New Zealand is free of SLF, but it is considered one of the most unwanted pests because of the high risk this insect is likely to pose to key horticultural industries, including kiwifruit, vines, apples and pears [5].

Both nymphs and adults of SLF feed on a broad spectrum of host plants by piercing the young stems and leaves and sucking out the phloem [6,7,8]. As this insect tends to feed in groups, the damage caused by feeding can result in withering or death of branches [9]. In addition to the physical damage it causes, the honeydew secreted by SLF can lead to sooty mold infestations [10]. In USA, SLF is reported to be a severe threat to various crops and ornamental plants, including fruit trees, ornamental trees, woody trees, and vines [10,11,12]. Similarly, in China, it is considered a significant pest of various crops, including kiwifruit [13,14]. Both nymphs and adults feed on the leaves, vines, and trunks of kiwifruit plants, leading to the growth of sooty mold [13,14]. Nymphs aggregate under the leaves and vines of kiwifruit, causing damage [14], whereas adults lay eggs on vines, trunks, fruits, and the support structure of the kiwifruit [13]. In some Chinese kiwifruit orchards, yield losses have been projected to reach 80–90% based on limited field observations [15].

Recent observations from invaded regions in USA indicate that SLF is difficult to control once established [16]. Currently, no effective biological control agents have been identified for SLF in these areas. Research on eradicating SLF focused on the effective use of insecticides, concluding that SLF is sensitive to broad-spectrum pyrethroids, organophosphates, and neonicotinoid insecticides [17,18]. It has been shown in ovicidal bioassays that only chlorpyrifos significantly increases egg mortality in SLF [17]. In China and South Korea, various insecticides, such as deltamethrin, fenitrothion, imidacloprid, clothianidin, thiacloprid [18], and etofenprox combined with diazinon, chlorpyrifos, etofenprox, and dinotefuran have been used to combat the nymphs [19]. In addition, lime sulfur owing to its prolonged efficacy, low propensity for resistance development, minimal residues, and environmental compatibility, has been shown to be effective and extensively utilized in the organic production of various fruits [20].

In New Zealand, bifenthrin combined with the penetrative agent Engulf have been shown to be effective in reducing the hatching rate of egg masses of other planthoppers such as passion vine hopper (*Scolypopa australis*), and chorus cicada (*Amphipsalta zelandica*) on kiwifruit vines [21]. Similarly, bifenthrin, thiacloprid, pyrethrum, and Spinosad, which have been tested against SLF, are approved for use against other known pests during specific phases of the kiwifruit cropping cycle [19]. Many of these insecticides are not registered for use in kiwifruit in New Zealand. Therefore, there is a need to understand whether these insecticides could be used at current New Zealand label rates as an eradication or long-term management tool to control SLF eggs and nymphs in kiwifruit in the event of an invasion. Since New Zealand has not been invaded by SFL yet, the direct testing of insecticide efficacy against this pest is currently unfeasible there. This study was conducted in China, where SLF is endemic and readily available, enabling evaluation of ovicidal and nymphicidal efficacy while adhering to New Zealand’s safety standards. Laboratory bioassays were performed under controlled conditions to minimize environmental variability, while field trials provided preliminary insights into the real efficacy, despite climatic differences between China and New Zealand that may lead to variations in insecticide performance (e.g., due to temperature and humidity effects) [22]. To date, no specialized biotypes of SLF have been reported. Biotypes are likely driven by genetics, population-genetic analyses by Lee demonstrated that all Korean and Japanese isolates share the same genotypes as populations from four different sites in China [23]. Indication that same biotype of SLF in original area East Asia, as well as in the new invasion region. Accordingly, results obtained from our study on a single endemic Chinese population should be broadly applicable to SLF populations elsewhere. In this study, three concentrations of bifenthrin and lime sulphur were initially evaluated as potential tools for controlling the SLF egg in the laboratory. Subsequently, bifenthrin, thiacloprid, and pyrethrum plus mineral oil were evaluated as potential insecticides against nymphs. Finally, the short-term knockdown efficacy and long-term residual activity of these insecticides against nymphs were evaluated through field trials.

## 2. Materials and Methods

### 2.1. Insecticides

We tested insecticides that are currently used by New Zealand kiwifruit growers and are listed in the Zespri crop protection standard (CPS) that are also commercially available in China. Specifically, bifenthrin with polyether-modified trisiloxane and lime sulphur were tested against SLF eggs. Natural pyrethrins with mineral oil, bifenthrin, and thiacloprid were tested against SLF nymphs. Details of the tested agrichemicals are provided in Table 1.

### 2.2. Laboratory Ovicidal Bioassays

Preliminary surveys indicated that SLF exhibits distinct host preferences at different developmental stages. To obtain sufficient specimens, the eggs or nymphs used for bioassays were collected from a single colony on the same host plant. In total, 176 SLF egg masses were collected from wild peach trees in Beijing, China on 18 March 2024. Egg masses were sent to an indoor laboratory, and each egg mass was kept individually in a Petri-dish (d = 5 cm) and held in incubator (23 °C).

Insecticide concentrations based on the product labels currently approved for use on kiwifruit in New Zealand were used. Egg masses were carefully measured with a steel ruler (10 cm) without touching the wax coating cover. Then area of egg masses was calculated by multiplying the length by the width. The results of egg mass area would be used to calculate the dose of agrichemicals based on the label. To simulate field conditions, the spraying method was employed to determine the efficacy of the insecticide. In New Zealand, the recommended active ingredient rate of bifenthrin is 0.0006 mg/cm^2^. The average area of each egg mass was measured as 4.0 ± 1.6 cm^2^. For a 4 cm^2^ egg mass, this means 0.0024 mg of active ingredient is needed. 0.125 mL of liquid from a 5 mL spray bottle would cover the 4 cm^2^ egg mass area from preliminary tested. A total of 19.2 µg active ingredient was needed per mL water (0.0024 mg/0.125 mL) to achieve the label dose.

In the formulation process of the insecticide, approximate ratios were utilized to mix the components (Table 2). All the insecticides were prepared from the highest concentration and then gradually diluted. Bifenthrin at the label concentration (19.2 μg/mL), as well as at 10 times and 100 times of this concentration, were prepared from the Chinese product (Table 1). Engulf (Polyether-modified trisiloxane) was added at a rate recommended by the manufacturer, mixed with bifenthrin, to achieve a final concentration of 0.1% Engulf. Lime sulfur was prepared at its labeled concentration (1.3 mg/mL) and at 10- and 100-times that concentration, using the same procedure as for bifenthrin (Table 1 and Table 2). In total, 10 treatments were evaluated (Table 3), plus a distilled water control were tested, and 16 replications were used for each treatment and control.

**Figure 1:**
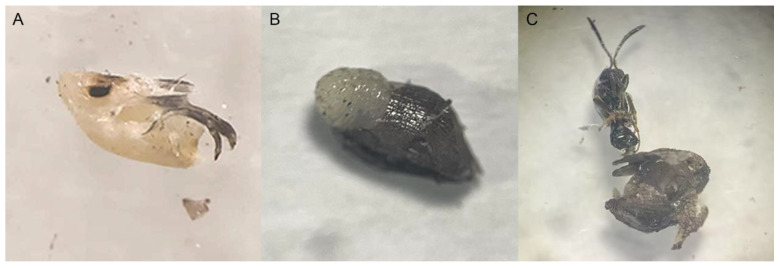
The anatomical identification in dead SLF eggs include (**A**) Nymphs of *Lycorma delicatula*, (**B**) pre-pupae of *Anastatus orientalis* and (**C**) Adult of *Anastatus orientalis*.

The mortality rate of egg was determined by dissecting unhatched eggs to exclude the effect of random parasitism by parasitoids. Dead individuals within the SLF egg masses were found through dissection. Based on morphological identification using mainly mouthparts characteristics, the dissected individuals were determined to be dead SLF nymphs (Figure 1A) or unhatched *Anastatus orientalis* parasitoids Yang (Figure 1B,C). The nymph mortality rate was calculated as the ratio of the number of dead nymphs found by dissection to the total number of nymphs (the number of hatched nymphs plus the number of dead nymphs obtained by dissection). Adjusted mortality was used to compare the activity differences of various insecticides against SLF nymphs.

### 2.3. Laboratory Nymph Bioassays

**Figure 2:**
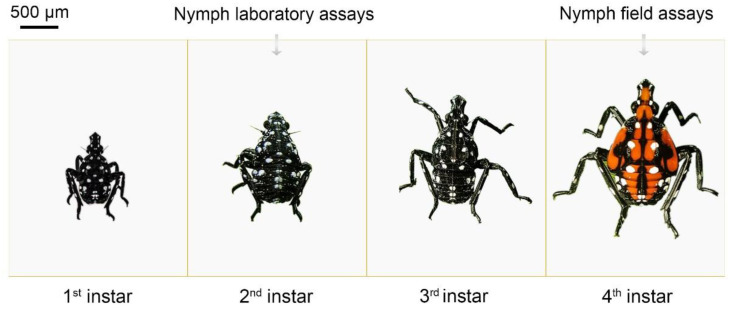
Nymph instar stages of SLF evaluated in laboratory and field cage bioassays.

**Figure A1:**
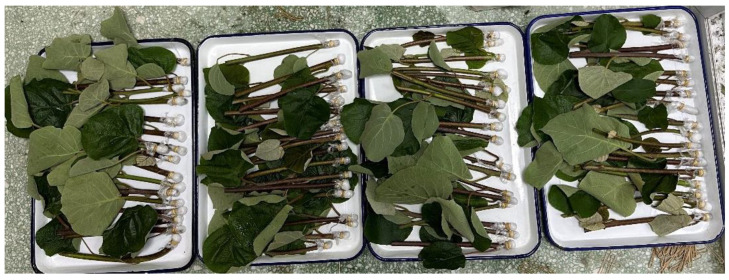
Hayward cuttings were prepared for testing insecticides residual efficacy.

**Figure A2:**
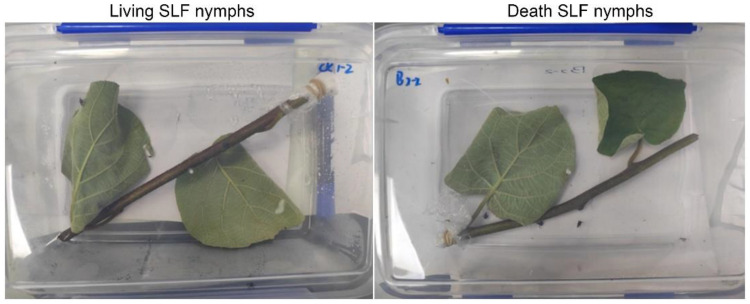
Living and dead SLF nymphs and cutting branches after 48 h of insecticide treatment in containers.

To ensure phenological synchrony between experimental timelines and the developmental stages of field SLF populations, second-instar SLF were selected as a representative of young nymphs for laboratory bioassays. On 14 May 2024, 288 s instar nymphs (Figure 2) were collected from tree of heaven and placed into a netted cage (60 × 60 × 60 cm, Mega View Science Co., Ltd., Taiwan, China) with plant cuttings. Insecticides and surfactants were accurately weighed using an analytical balance (±0.001 g) for solids and/or precisely measured using adjustable micropipettes (±1 µL) for liquids. Due to differences in the concentrations of chemical insecticides between China and New Zealand, the application rates of insecticides were adjusted to match the label rates of products currently registered for use on kiwifruit in New Zealand. Bifenthrin 0.4 mL/L, thiacloprid 0.384 g/L, pyrethrins 4.6 mL/L (95%) combined with mineral oil 8.490 g/L (5%) were prepared referring to the recommended label rate. Thirty milliliters of prepared insecticides were sprayed on 30 cm untreated kiwifruit (Hayward) cuttings, each with two leaves. The bottom of the cuttings was covered with cotton soaked in water and sealed with parafilm (Figure A1). After the insecticide dried, the cuttings were placed individually in 36.8 × 25 × 11 cm plastic deli containers (Anhui Hualong Plastic Co., Hefei, China) (Figure A2). Four 2nd instar SLF nymphs were placed into each container, and mortality was checked at 24 h and 48 h for each assessment. 18 replications for each treatment and control (distilled water spray) were used.

### 2.4. Nymph Field Bioassays

**Figure A3:**
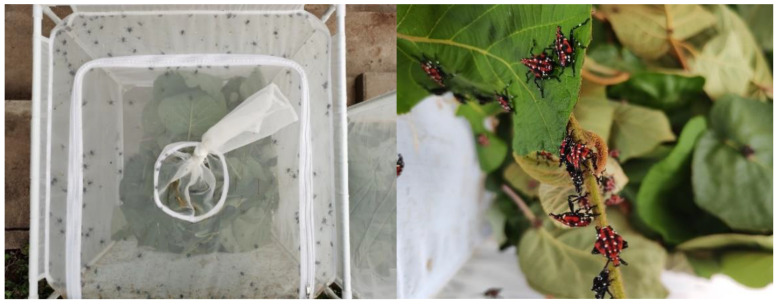
Fourth instar SLF nymphs were collected the night before for testing the residual efficacy of insecticides test in the field. On the left was the third instar nymph, and on the right was the fourth instar nymph.

As the terminal nymphal stage in SLF, fourth instars theoretically confer the higher pesticide resistance; consequently, fourth-instar SLF nymphs were selected for field bioassays. On 7 June 2024 the efficacy of insecticides against nymphs were carried out in field trials at Mei County, Shanxi, China. In total, 400 4th instar nymphs (Figure 2) were collected from nearby untreated Kiwifruit vine and held overnight in netted cages (60 × 60 × 60 cm) with kiwifruit cuttings for feeding (Figure A3). Ten kiwifruit trees were randomly selected; four vines were chosen and marked with colored tape from each tree for bioassays.

**Figure A4:**
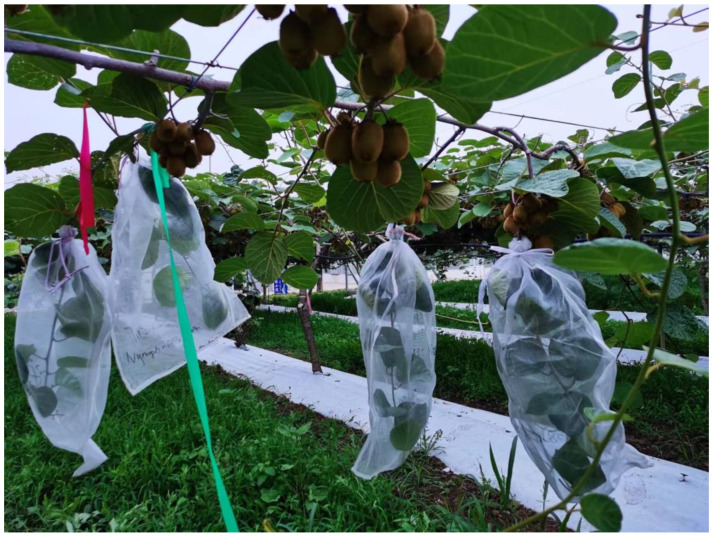
The efficacy of three insecticides treatments and a control were tested in the field.

All insecticides and distilled water (control) were sprayed in the morning to minimize wind speed and insecticide drift by spray bottles (#GR-1000, Xuzhou Gongrui Commercial and Trading Ltd., Xuzhou, China). Insecticides and surfactants were prepared following the same protocol described above for the lab nymph experiments. One hundred milliliters of insecticides were applied to thoroughly wet vine (around 100 cm) of the kiwifruit tree, including the underside of the leaves which were taped before, but not until runoff. Once the vine was dry, a nylon mesh-sleeve cage (100 × 80 cm, 60 μm) was used to cover the vine and ten SLF nymphs were added to the cages (Figure A4). The number of dead nymphs was recorded 48 h later, and then all insects and cages were removed from the kiwifruit vine for normal weathering of the chemical residues due to rain or sunlight. On the 7th and 14th days after the initial treatment date, a clean cage and ten newly collected 4th instar nymph were placed on each individual vine, and nymphal mortality was measured 48 h after this exposure. 10 replications for each treatment and control (distilled water spray) were used.

### 2.5. Data Analysis

When the mortality rate of the control group was greater than 5%, the efficacy of insecticides was evaluated using the adjusted mortality rates (MR) [24]. MR was calculated using the formula: MR (%) = (Mt − Mc)/(1 − Mc) × 100%. In this formula, Mt represents the mortality rate of pests observed in an insecticide treatment, and Mc represents the mortality rate observed in the control group. Adjusted mortality is a control-normalized proportion rather than a simple binary outcome, making conventional binary analyses inappropriate. Therefore, following the approach of [25], we evaluated its statistical significance using a Poisson distribution.

In all bioassays, adjusted mortality for each treatment was analyzed with a generalized linear model (GLM) assuming a Poisson error distribution, and Sidak-corrected pair-wise contrasts were employed for post hoc multiple comparisons. All statistical analyses were performed using SPSS Statistics 27.

## 3. Results

### 3.1. Efficacy of Laboratory Ovicidal Bioassays

Significant differences in the adjusted mortality of SLF eggs were detected and these were driven by both the dose–response (x^2^ = 31.02, df = 2, *p* < 0.001) and the type of insecticide applied (x^2^ = 6.57, df = 2, *p* < 0.05). Additionally, there was a significant interaction between insecticide types and concentrations (x^2^ = 41.47, df = 8, *p* < 0.01). Significant differences in adjusted mortality of SLF eggs were observed among various treatments (x^2^ = 49.03, df = 9, *p* = 0.806) (Table 3). The highest adjusted egg mortality (71.8 ± 8.5%) was achieved with bifenthrin at 100 times the label concentration, followed by bifenthrin combined with Engulf at the same rate (61.3 ± 8.5%), and lime sulphur at 100 times the label concentration (59.1 ± 9.3%) (Table 3). However, no significant differences were observed among these three treatments. The adjusted mortality was 34.6 ± 8.9%, 36.7 ± 8.2%, and 13.3 ± 7.9% caused by label dose of bifenthrin, bifenthrin + Engulf, and lime sulphur, respectively. Engulf, a polyether-modified trisiloxane adjuvant, did not significantly enhance the efficacy of bifenthrin against SLF eggs at any of the three concentrations tested (Table 3). The average number of eggs per egg mass was 31.9 ± 0.9. Dissection results indicate that the natural rate of parasitism was 23.8 ± 2.3% (number of parasitized eggs/total number of eggs within an egg mass), with a maximum parasitism rate within a single egg mass reaching 100% (number of parasitized eggs in a single egg mass/total number of eggs in that egg mass). Additionally, the parasitism rate of egg masses was 69.1% (number of parasitized egg masses/total number of egg masses). The parasitism was entirely carried out by one parasitoid species, identified as *Anastatus orientalis* (Hymenoptera: Eupelmidae) by Peng [26].

### 3.2. Efficacy of Laboratory Nymph Bioassays

Significant differences in adjusted mortality rates were observed among the various insecticide treatments at 24 h assessments, (x^2^ = 13.54, df = 2, *p* < 0.001) (Table 4). Bifenthrin and thiacloprid demonstrated high efficacy, achieving adjusted mortality rates of 93.7 ± 3.7% and 92.1 ± 3.1% at 24 h, respectively, with no significant difference between them. However, both treatments were significantly more effective than the pyrethrins combined with mineral oil treatment, which exhibited a mortality rate of 69.8 ± 5.9%.

Similarly, at the 48 h assessments, significant differences in adjusted mortality rates were observed among the various insecticide treatments (x^2^ = 12.06, df = 2, *p* < 0.01). Thiacloprid and bifenthrin both achieved mortality rates of 100.0 ± 0.0%, significantly higher than those of the pyrethrins combined with mineral oil treatment, which reached 83.3 ± 5.6%.

### 3.3. Efficacy of Nymph Field Bioassays

Bifenthrin is a contact insecticide, whereas thiacloprid acts as a systemic insecticide. Consequently, we documented the weather variations throughout the testing procedure. The field experiment was conducted over a two-week period from 7 to 20 June 2024. During this entire testing period, there was one day of thunderstorms within the first 48 h, two days of thunderstorms within the first 7 days, and a total of three days of thunderstorms and one day of moderate rain. Additionally, the average high temperature was 34.9 °C, with an average low of 19.5 °C. In insecticide residue bioassays, treatments with different insecticide types across three residual periods significantly influenced the adjusted mortality of SLF (x^2^ = 36.17, df = 2, *p* < 0.01). The residual period post-treatment significantly affected mortality rates within each insecticide type (x^2^ = 43.08, df = 2, *p* < 0.01). Additionally, there was a significant interaction between insecticide type and residual period (x^2^ = 82.37, df = 8, *p* < 0.01).

On the same day of pesticides application (0 day), significant differences in adjusted mortality were observed among the treatments (x^2^ = 15.44, df = 2, *p* < 0.001) (Table 5). Bifenthrin and thiacloprid treatments resulted in adjusted mortality rates of 100 ± 0.0% and 98.9 ± 1.1%, respectively, with no significant difference between them. However, both treatments were significantly more effective than the pyrethrins combined with mineral oil treatment. At a residual period of 7 days, significant differences in adjusted mortality were observed among the treatments (x^2^ = 15.78, df = 2, *p* < 0.001) (Table 5). Thiacloprid and bifenthrin resulted in adjusted mortality rates of 72.8 ± 5.9% and 52.4 ± 7.7%, respectively, with no significant difference between them. However, both treatments were significantly more effective than the pyrethrins plus mineral oil treatment. At a residual period of 14 days, significant differences in adjusted mortality were also observed among the treatments (x^2^ = 13.69, df = 2, *p* < 0.001) (Table 5). Thiacloprid acts as a systemic insecticide achieved the highest adjusted mortality rate of 46.7 ± 7.2%, significantly exceeding the mortality rates caused by contact insecticide which bifenthrin and the pyrethrins plus mineral oil treatment. No significant difference was detected between bifenthrin and the pyrethrins plus mineral oil treatment.

## 4. Discussion

The invasion of SLF is becoming increasingly problematic globally. In this study, we first assessed the ovicidal efficacy of various insecticides at the New Zealand label rate and conducted tests at 10× and 100× concentrations. Bifenthrin at 100 times the label concentration demonstrated the best ovicidal efficacy, with an adjusted mortality rate of 71.8 ± 8.5%. Subsequently, laboratory and field tests were conducted to assess nymphicidal efficacy, revealing that bifenthrin and thiacloprid at label concentration had exceptionally high short-term mortality rates. Furthermore, thiacloprid at label concentration showed stronger residual activity, with adjusted mortality rates of 72.8% ± 5.9% and 46.7 ± 7.2% at 7 and 14 days post-treatment, respectively.

In laboratory ovicidal bioassays, we dissected all the overwintering SLF egg masses to check the fate of each egg. There were two scenarios observed in the eggs by dissected, one was the dead SLF (nymph/egg) and another was the parasitoids. In our testing, the SLF eggs were attacked by parasitoid, identified as *A. orientalis*, before we collected them in the field. This is consistent with previous research, *A. orientalis* parasitized SLF at a high rate in China [27]. The importance of dissection in accurately evaluating the true efficacy of ovicidal tests should be emphasized, as prior to dissection, the insecticide efficacy tests against SLF eggs yielded extremely unsatisfactory results. Some insecticides treatments even showed mortality rates lower than the control [25]. Moreover, the effects of the insecticides examined in the present study on egg parasitoids have not yet been investigated. Future research should assess the safety of these insecticides to egg parasitoids in order to determine their compatibility with IPM programs in China or New Zealand.

The 100 times label rate for bifenthrin, bifenthrin combined with engulf, and lime sulphur displayed the highest efficacy. However, these pesticides performed poorly at label concentrations. These suggest that, under current label concentrations, neither bifenthrin nor lime sulphur is likely to be adequate for controlling SLF eggs. These findings are consistent with earlier studies, where only chlorpyrifos achieved 100% mortality, and few other products exhibited significant ovicidal activity [17,25]. The limited activity of most products is likely attributable to the waxy layer covering the egg mass surface, which confers substantial protection to the developing embryo [28]. Nevertheless, considering prior surveys indicating that SLF exhibits a univoltine life cycle [29], high-concentration insecticides (at 100 times label concentration) could serve as a viable contingency measure. Specifically, ovicidal interventions targeting SLF eggs should be initiated during winter, when high concentrations can be applied without impacting fruit production, and the insecticides undergo slow degradation under field conditions. In the context of severe SLF invasions, where rapid containment is paramount, such applications may be feasible under emergency eradication strategy. However, this approach must carefully weigh potential environmental contamination risks, including soil and water residues. Future studies should evaluate degradation kinetics and non-target impacts in winter field trials to refine these strategies, ensuring they align with sustainable pest management practices.

Interestingly, in our experiment, adding Engulf did not significantly enhance bifenthrin’s efficacy, despite previous studies showing that Engulf significantly increased bifenthrin’s penetration and lethality in passion vine hopper and chorus cicada eggs [21]. Possible reasons could be the specific structure of SLF egg masses hindering Engulf’s penetration, or differences in environment, climate, or pH levels.

In both laboratory and field experiments, three insecticides were tested for their lethal effects on 2nd and 4th instars SLF nymphs on kiwifruit shoots. The results indicated that bifenthrin and thiacloprid had very high short-term efficacy, achieving nearly 100% mortality within 48 h for both instars. These findings are consistent with previous reports on the insecticidal efficacy of bifenthrin and thiacloprid against SLF nymphs, indicating that even at the fourth instar, SLF nymphs do not develop additional resistance to these agents [17,18]. However, while the combination of pyrethrins and mineral oil resulted in a relatively high adjusted mortality rate for 2nd instar SLF nymphs, it performed poorly against 4th instar nymphs, suggesting that older instars may have higher tolerance to this insecticide.

In residue activity tests, pyrethrins combined with mineral oil lost most of its efficacy. Pyrethrins are the primary active components of pyrethrum, therefore, this experiment indirectly supports the conclusion that pyrethrum is less effective against nymphs in some tests [30]. Bifenthrin maintained substantial residual ovicidal activity against SLF nymphs at 7 days post-application, comparable to that of thiacloprid. However, by day 14, it had lost nearly all efficacy. This finding contrasts with earlier tests on grapevines, which reported that bifenthrin maintained high activity against nymphs through day 14 [25,31]. The discrepancy may be attributed to differences in the nymphal instars tested, different tested plants, the residue insecticides exposure environment. Firstly, differences in the developmental stage of nymphs tested may account for these observations [32]. Then, the specialized chemical composition and structure of different plant surfaces may influence pesticide insecticide adsorption and persistence [33]. Most importantly, the residual activity of insecticides is modulated by environmental factors [22]. Given that thiacloprid is systemic [34], whereas bifenthrin acts by contact [35], the residual efficacy of bifenthrin is expected to be more markedly diminished by rainfall during the experimental period. Among the insecticides tested, Thiacloprid exhibited the remarkable efficacy on kiwifruit shoots. It maintained significantly higher activity levels than the other two insecticides at day 14. This indicates that thiacloprid at label concentration could be the best control tool against SLF nymphs at kiwifruit vines. The residual effects of insecticides are crucial in the management of SLF, as short-term chemical treatments, while capable of eliminating most SLF nymphs and adults within orchards, leave these areas vulnerable to reinvasion by surrounding SLF populations. Furthermore, SLF exhibits long-distance migration capabilities [36], which may result in persistent pest incursions into orchards.

Given the climatic disparities between New Zealand and China which may result in differences in insecticide efficacy [22] the reasults of field trial from Chinese regions just provided valuable guidance for practical applications. Moreover, the laboratory and field experiments utilized second- and fourth-instar SLF nymphs, respectively; therefore, the resulting data should be considered applicable only to these specific developmental stages. Future studies should extend these investigations to multi-site climate simulation experiments and assess the performance of label-rate insecticides across the full range of SLF nymphal instars.

We highlight the critical importance of implementing integrated pest management (IPM) protocols in kiwifruit orchards [19,37,38]. This could include removing prefered host of SLF, *A. altissima* (tree-of-heaven) near orchards [19]; pruning and burning egg-laying branches to reduce pest sources; applying whitewash before overwintering egg-laying to stop adults from climbing and laying eggs and using light traps to capture during peak adult activity in summer [39]. These physical control methods, combined with biological control measures, are crucial, particularly in addressing SLF egg suppression limitations of chemical methods. *Anastatus orientalis*, found in Northern China, is an important natural enemy of SLF eggs [40]. Recent surveys across four provinces in China recorded a maximum egg mass parasitism rate of 69%, with the highest individual egg parasitism rate at 33% [40]. In this experiment, *A. orientalis* exhibited a natural parasitism rate of 23.8% ± 2.3%. Given its high parasitism efficiency, research is being conducted in Korea and the United States to explore it as a potential biocontrol agent [41]. The potential for biological control is substantial, considering that *Antheraea pernyi* Guérin-Méneville, used experimentally in commercial applications, can serve as its host [41]. However, given that this paraistoid has not been detected locally in New Zealand, the inadvertent introduction of *Antheraea* could pose transboundary ecological risks. In the future, we recommend identifying host-specific parasitoids or locally occurring natural enemies for effective pest management in New Zealand.

## 5. Conclusions

This study selected a few insecticides approved for use against various pests during specific phases of the kiwifruit cropping cycle in New Zealand, and evaluated their efficacy in controlling SLF eggs and nymphs on kiwifruit vines. Bifenthrin at 100 times the label rate showed the highest efficacy for eggs, while thiacloprid demonstrated the most effective and long-lasting control for nymphs. Despite bifenthrin’s effectiveness, its high concentration poses environmental risks, suggesting it should be used as last-resort measure such as emergency eradication measures. The addition of Engulf did not significantly enhance bifenthrin’s efficacy against eggs. Taken together, these findings highlight the importance of using these effective insecticides in combination with biological control and physical methods as part of an integrated pest management strategy to achieve optimal sustainable management of SLF.

## Figures and Tables

**Table 1:** List of Chinese pesticide products with details on formulation, toxicity, and manufacturer.

Pesticide Active Ingredient	Technical Grade in CN	Formulation	Toxicity	Company
Bifenthrin	25 g/L	Emulsifiable concentrate	II*	Shandong Shibang Agrochemical Ltd. (Heze, China)
Lime sulphur	29%	Aqueous solution	III*	Sichuan Yibin Chuanan Gaoke pesticide Ltd. (Yibin, China)
Engulf (Polyether-modified trisiloxane)	99%	Aqueous solution	III*	Shandong Lulong Biotechnology Ltd. (Weifang, China)
Thiacloprid	25%	Water dispersible granule	II*	Qingdao Odyssey Biotechnology Ltd. (Qingdao, China)
Pyrethrins	1.5%	Emulsion in water	II*	Chengdu New Sun Crop Science Ltd. (Chengdu, China)
Mineral oil	97%	Emulsifiable concentrate	III*	Shanghai Hulian Bio-Pharmaceutical (Xiayi) Ltd. (Shanghai, China)

Note: II* stands for “Moderately toxic”. III* stands for “Low toxicity”.

**Table 2:** The calculations of insecticide concentrations as specified by New Zealand labeling and the corresponding actual concentrations of those labeled agents.

Types Insecticides/ Accessory Ingredient	Bifenthrin	Lime Sulphur
Recommend active ingredient rate per cm^2^ in NZ	0.0006 mg	0.042 mg
The dosage of CN product (Label solution) (/100 mL water)	76.8 µL (80 µL)	463.2 µL (500 µL)
Label rate	19.2 µg/mL (20 µg/mL)	1.3 mg/mL (1.4 mg/mL)

Note the values in “()” indicates the actual dosage of CN product utilized during the preparation of the Label solution.

**Table 3:** Adjusted mortality of SLF eggs exposed to different concentrations of insecticides.

Treatment (Active Ingredient and Concentration)	Rate	Adjusted Mortality (%)
Bifenthrin at label	19.2 µg/mL	34.6 ± 8.9 ^de^
Bifenthrin at 10× label	192 µg/mL	31.5 ± 7.9 ^de^
Bifenthrin at 100× label	1.9 mg/mL	71.8 ± 8.5 ^a^
Bifenthrin + Engulf at label	19.2 µg/mL	36.7 ± 8.2 ^cd^
Bifenthrin + Engulf at 10× label	192 µg/mL	40.3 ± 8.5 ^bc^
Bifenthrin + Engulf at 100× label	1.9 mg/mL	61.3 ± 8.5 ^ab^
Engulf label	990 µg/mL	16.1 ± 10.4 ^def^
Lime sulphur at label	1.3 mg/mL	13.3 ± 7.9 ^ef^
Lime sulphur at 10× label	13.4 mg/mL	17.4 ± 8.9 ^def^
Lime sulphur at 100× label	134.3 mg/mL	59.1 ± 9.3 ^ab^

Note: The control group exhibited a mortality rate of 15.5% ± 5.2%. Means labeled with the same lowercase letter do not differ significantly (*p* > 0.05).

**Table 4:** Adjusted mortality of SLF nymphs caused by three insecticides 24 h and 48 h after treatment.

Treatment (Active Ingredient)	Adjusted Mortality (%)	
	24 h	48 h
Thiacloprid	92.1 ± 3.1 ^a^	100.0 ± 0.0 ^a^
Bifenthrin	93.7 ± 3.7 ^a^	100.0 ± 0.0 ^a^
Pyrethrins + Mineral oil	69.8 ± 5.9 ^b^	83.3 ± 5.6 ^b^

Note: The control group exhibited a mortality rate of 15.6% ± 10.7%. Means labeled with the same lowercase letter do not differ significantly (*p* > 0.05).

**Table 5:** Residual activity of different insecticides on adjusted mortality of SLF nymphs after 48 h exposure to sprayed foliage at different residual periods (0, 7, or 14 days after treatment).

Treatment (Active Ingredient)	Adjusted Mortality (%)		
Residual period	0 day	7 day	14 day
Bifenthrin	100.0 ± 0.0 ^a^	52.4 ±7.7 ^a^	13.3 ± 4.3 ^b^
Thiacloprid	98.9 ± 1.1 ^a^	72.8 ± 5.9 ^a^	46.7 ± 7.2 ^a^
Pyrethrins + Mineral oil	41.5 ± 12.5 ^b^	19.8 ± 6.8 ^b^	12.2 ± 3.9 ^b^

Note: Insects were freshly collected prior to each assessment at 0, 7, and14 days post-treatment. The control group exhibited mortality rates of 9.0 ± 2.3%, 8.0 ± 3.6%, and 10.0 ± 3.3% at these respective time points. Within the same column, means labeled with the same lowercase letter are not significantly different. (*p* > 0.05).

## Data Availability

The data presented in this study are available on request from the authors.
